# Endothelial cell alterations in capillaries of adipose tissue from patients affected by lipedema

**DOI:** 10.1002/oby.24244

**Published:** 2025-03-12

**Authors:** Sandro Michelini, Stefania Greco, Nicola Vaia, Valeria Puleo, Pamela Pellegrino, Angelica Di Vincenzo, Serena Michelini, Karen L. Herbst, Gaia Goteri, Tonia Luca, Sergio Castorina, Antonio Giordano, Pasquapina Ciarmela, Saverio Cinti

**Affiliations:** ^1^ Vascular Diagnostics and Rehabilitation Service Marino Hospital Rome Italy; ^2^ Physical Medicine and Rehabilitation San Giovanni Battista Hospital Rome Italy; ^3^ Department of Experimental and Clinical Medicine, Center of Obesity Università Politecnica delle Marche (Polytechnic University of Marche) Ancona Italy; ^4^ Plastic, Reconstructive and Aesthetic Surgery European Hospital Rome Italy; ^5^ Department of Science and Public Health Catholic University Policlinico Gemelli Rome Italy; ^6^ The Roxbury Institute Tucson Arizona USA; ^7^ Department of Biomedical Sciences and Public Health, Section of Pathological Anatomy and Histopathology Università Politecnica delle Marche (Polytechnic University of Marche) Ancona Italy; ^8^ Department of Medical, Surgical Sciences and Advanced Technologies "G.F. Ingrassia" University of Catania Catania Italy; ^9^ Istituto di Ricovero e Cura a Carattere Scientifico (Scientific Institute for Research, Hospitalization and Health Care), Istituto Nazionale di Ricovero e Cura per Anziani (National Institute of Hospitalization and Care for the Elderly) (IRCCS/INRCA) Ancona Italy

## Abstract

**Objective:**

This study aimed to evaluate adipose tissue of lipedema patients.

**Methods:**

Gluteo‐femoral (affected area) and interscapular (nonaffected area) adipose tissue from 10 lean patients affected by lipedema stage 1 to 2 was studied and compared with tissue from 10 patients with obesity and 12 lean patients.

**Results:**

The main features were alterations of capillaries with wall thickening (*p* ≤ 0.0001), endothelial and pericyte hyperplasia (*p* = 0.03 and *p* = 0.004), hypodense areas in basal membrane, and endothelial degeneration with exfoliation of degenerated cells into the capillary lumen. Adipocytes were larger (hypertrophic) in affected (*P* ≤ 0.0001) and nonaffected (*p* = 0.0003) areas compared with those with obesity and who were lean (both *p* ≤ 0.0001). Frequently the cytoplasm of adipocytes contained massive deposition of calcium crystals as revealed by Von Kossa staining (*p* = 0.023) and electron microscopy. CD68 immunoreactive macrophages were more abundant in affected areas (*p* = 0.005), and their number was similar to that found in fat from patients with obesity (*p* = 0.17). Despite adipocyte hypertrophy and inflammation, lack of the healthy marker perilipin‐1 and the presence of crown‐like structures were only rarely seen, while they were quite frequent in patients with obesity.

**Conclusions:**

Our data support the idea that cell alterations happen in the early stages of adipocyte development (endothelium/pericyte) in the adipose organ of women affected by lipedema.

**Study Importance**

**What is already known?**

- Lipedema is a female‐specific pathology of subcutaneous fat with unknown pathogenesis.
- It is characterized by an abnormal symmetrical increase in subcutaneous adipose tissue of the limbs, with almost no involvement of the trunk, hands, or feet, resulting in a “bracelet” effect of tissue at the ankle and wrist.
- Different from patients with obesity, the abnormal increase of adipose tissue causes symptoms such as pain, tenderness, increased vascular fragility, arthritis, and easy bruising.

**What does this study add?**

- The study shows evidence for specific alterations of capillaries with wall thickening, endothelial/pericyte hyperplasia, and endothelial cell degeneration by light and electron microscopy.
- Adipocytes were larger in affected and nonaffected areas of lipedema adipose tissue compared with those in patients with obesity or lean patients. Frequently, the cytoplasm of adipocytes contained massive deposition of calcium crystals.
- Although that CD68 immunoreactive macrophages were more abundant in affected lipedema areas, crown‐like structures were only rarely seen.

**How might these results change the direction of research or the focus of clinical practice?**

- Our data suggest a role of endothelial cell alterations in the pathogenesis of lipedema offering new insight for further research both for diagnostic and therapeutic targets.
- The endothelial hyperplasia with endothelial degeneration and vascular invasion could offer a new tool for the diagnosis of lipedema patients.
- Endothelial cell markers should be very high in patients with lipedema, possibly representing novel and early blood markers of lipedema.

## INTRODUCTION

Lipedema is a pathology of still unknown origin that mainly affects women of young age, often by the third decade of their life. It is characterized by an abnormal symmetrical increase in subcutaneous adipose tissue of the limbs, with almost no involvement of the trunk, hands, or feet, resulting in a “bracelet” effect of tissue at the ankle and wrist [1, 2, 3]. Different from patients with obesity, in patients with lipedema, the abnormal increase of adipose tissue causes symptoms such as pain, tenderness, increased vascular fragility, arthritis, and easy bruising [4, 5, 6]. Adipose tissue of patients suffering from lipedema is altered in defined sites of subcutaneous fat [4, 7]; however, a specific morphological characterization comparing the histology and electron microscopy of affected (limb) and nonaffected (trunk) areas has not yet been reported, to our knowledge. Furthermore, because the most common pathology with fat accumulation is obesity, we compared data of lipedema patients with those of 10 female patients affected by obesity and those of 12 lean patients.

Our data support the hypothesis that endothelial cell alteration is a key feature in the histopathology of lipedema.

## METHODS

### Biopsy

We studied 20 biopsies from 10 normal‐weight female patients affected by lipedema. Participants were determined to have lipedema based on the criteria defined by the standard of care for lipedema in the United States [8] and were selected among about 300 patients with this pathology in order to have a case series of patients mainly in stage 1 to 2, types II and III (see clinical data in Table 1) and in order to reduce, as much as possible, the risk of pathogenic overlaps due to obesity.

**TABLE 1:** Clinical data of patients with lipedema and obesity and lean controls.

Lipedema patients				
Patient	Diagnosis	BMI, kg/m^2^	Age, y	Note
1	Type II and IV, stage 2	25	54	Hashimoto's thyroiditis and hypertension
2	Type III, stage 2	21.9	19	Family history for Hashimoto's thyroiditis
3	Type III and IV, stage 2	24.9	59	Hypothyroidism, labile hypertension, mild hypercholesterolemia, and paroxysmal atrial fibrillation
4	Type III and IV, stage 2	25	41	Family history for type 2 diabetes, hypothyroidism, and hypovitaminosis D
5	Type II and IV, stage 2	24.7	57	Left breast quadrantectomy for K + chemotherapy and radiotherapy, cervical conization for K in situ, and osteopenia
6	Type II and IV, stage 2	25	40	Adrenal gland hyperplasia, ovarian micropolycytosis, and family history for type 2 diabetes and thyroid disease
7	Type III, stage 1	22.6	17	Edema of the lower limbs from puberty with paresthesia and dysesthesia and the appearance of “livedo” in the cold season
8	Type III, stage 1	23.8	22	Lower limb edema from puberty
9	Type II and IV, stage 1	24.8	43	Family history for thrombophilia and type 2 diabetes
10	Type III and IV, Stage 3	24.8	41	Family history for type 2 diabetes

Lean patients		
Patient	BMI, kg/m^2^	Age, y
1	24.8	49
2	27.3^a^	55
3	24.2	66
4	20.8	47
5	25	39
6	18.7	31
7	18.8	43
8.	24.8	17
9	24.6	29
10	24.6	23
11	25	38
12	19.6	39

Patients with obesity		
Patient	BMI, kg/m^2^	Age, y
1	50	38
2	41	27
3	40	46
4	40	41
5	43.25	53
6	43	32
7	48.28	46
8	43.9	56
9	42.8	52
10	43.6	54

^a^ Lean as determined by waist circumference and visually evident hypertrophic muscle apparatus.

Two punch biopsies were obtained from each patient: one from the subcutaneous adipose tissue in an area affected by lipedema (limbs) and one from the subcutaneous adipose tissue in the interscapular region (control site). The interscapular region was chosen as a control because lipedema rarely affects the subcutaneous fat of the trunk, which has been widely recognized [1, 8]. Additionally, this site was selected to minimize the aesthetic impact of the biopsies (Table 1).

We also analyzed subcutaneous fat from 10 female patients with subcutaneous obesity who were matched for age (Table 1). Abdominal subcutaneous fat biopsies from 12 patients undergoing cholecystectomy were used as lean controls (Table 1). All patients examined had no metabolic alterations, as shown by normal levels of metabolic markers in the blood (data not shown).

### Light microscopy

Formalin‐fixed tissue samples were embedded in paraffin, and 5‐μm sections were cut for hematoxylin & eosin (H&E) staining, histochemistry, and immunohistochemistry.

### Histochemistry

In order to evaluate the presence of calcium, the Von Kossa histochemical staining (Bio‐Optica) was used. The tissue composition evaluation was performed using the Bio‐Optica kit (Masson's trichrome stain with light green) according to the manufacturer's instructions. Four additional stains were used: Weigert's ferric hematoxylin for nuclei, picric acid for erythrocytes, a mixture of acid dyes (acid fuchsin “ponceau de xylidine”) for the cytoplasm, and light green for the collagen [9, 10].

Calcium deposition was assessed through morphometric analysis using Von Kossa‐stained slides. For each fat biopsy, 10 images were captured at 40× magnification. The percentage of the adipocyte perimeter that was positively stained was calculated using ImageJ software (National Institutes of Health).

Fibrosis was evaluated through morphometric analysis using Masson's trichrome‐stained slides. For each biopsy, 10 images were captured at 40× magnification, and the percentage of collagen deposition around the adipocyte perimeter was quantified using ImageJ.

### Immunohistochemistry

Tissue sections were stained with markers for macrophages (CD68, Dako/Agilent Technologies), endothelial cells (CD31, M0823, Dako/Agilent Technologies), lymphatic vessels (D2‐40, BioLegend), perilipin‐1 (PLIN1; Cell Signaling Technology), and cell‐proliferating marker (Ki‐67, GA62661‐2, Dako/Agilent Technologies) counterstained with hematoxylin; positive and negative controls were included and compared.

### Transmission electron microscopy

For the ultrastructural study, the biopsies were cut into fragments ~1 mm^3^ in size and immersed overnight in a fixative solution containing 2% glutaraldehyde and 2% paraformaldehyde in 0.1M phosphate buffer, pH 7.4. The samples were then rinsed with phosphate buffer, post‐fixed in 1% osmium tetroxide (OsO₄) for 60 min at 4°C, dehydrated in acetone, and embedded in Epon resin. Thin sections were prepared using an MTX ultramicrotome (Electron Microscopy Sciences), and 80‐nm sections were counterstained with lead citrate and examined using a Philips CM10 transmission electron microscope (TEM) operating at 100 kV.

For each biopsy, three to five semithin sections stained with toluidine blue were first observed. Thin sections from two to three selected areas were then obtained using the MTX ultramicrotome, stained with lead citrate, and analyzed via TEM.

### Adipocyte size

All images were captured at 20× magnification using the Nikon Eclipse E600 light microscope. A total of 100 adipocytes were measured in five different fields per participant from H&E‐stained histological sections as the mean cell area (in microunits squared).

Adipocyte area analysis was performed with ImageJ.

### Capillary vessels analysis

For evaluation of capillary wall thickness, capillaries were identified at 60× magnification in 10 fields in Masson's trichrome‐stained sections and analyzed using ImageJ. Masson's trichrome staining reveals the presence of the extracellular matrix around capillary vessels, which differ from arterioles because the capillary walls are characterized by a lack of muscular and elastic fibers [11]. As an additional specific criterion, we considered blood vessels with a diameter between 5 and 10 μm, i.e., in the normal range of capillaries [12, 13]. We used the same sections for investigation of the number of nuclei of endothelial cells and the number of pericytes per capillary.

### Inflammation and presence of crown‐like structures

In order to evaluate the number of macrophages and the presence of crown‐like structures (CLS), we used immunohistochemistry with CD68 (Dako/Agilent Technologies) antibodies. All images were captured at 20× magnification. In order to verify whether the macrophages organized to form CLS, the number of CLS was counted in all images and the percent density of CLS/10^2^ adipocytes was counted with ImageJ.

### Statistical analysis

All statistical analyses were conducted using GraphPad Prism (version 9.5.0, GraphPad Software). The results are displayed as minimum/maximum box and whisker plots, with the median indicated by a solid horizontal line within each box.

In order to evaluate differences between the areas affected and not affected by lipedema, we employed ANOVA, nonparametric ANOVA, and paired *t* tests. For multiple comparisons, the Siegel‐Tukey test and the Holm‐Sidak test were used. In figures, statistical comparisons are represented by a pound hash symbol (#) for comparisons with lipedema nonaffected areas, whereas an asterisk (*) indicates comparisons with lipedema‐affected areas. Statistical significance is denoted as follows: *p < 0.05; **p < 0.01; ***p < 0.001; ****p < 0.0001; #*p* < 0.05; ##*p* < 0.01; ###*p* < 0.001; and ####*p* < 0.0001.

### Ethical approval

This study protocol was approved and performed following the guidelines and regulations of the Local Ethics Committee of Marche Region (CERM), Prot. 2020 385 (patients with obesity or lean patients). For the study of biopsies of subcutaneous adipose tissue affected by lipedema, all methods were approved and performed following the guidelines and regulations of the Ethics Committee of Bolzano, Prot. 0241391‐BZ. All participants signed the written informed consent.

## RESULTS

**FIGURE 1:**
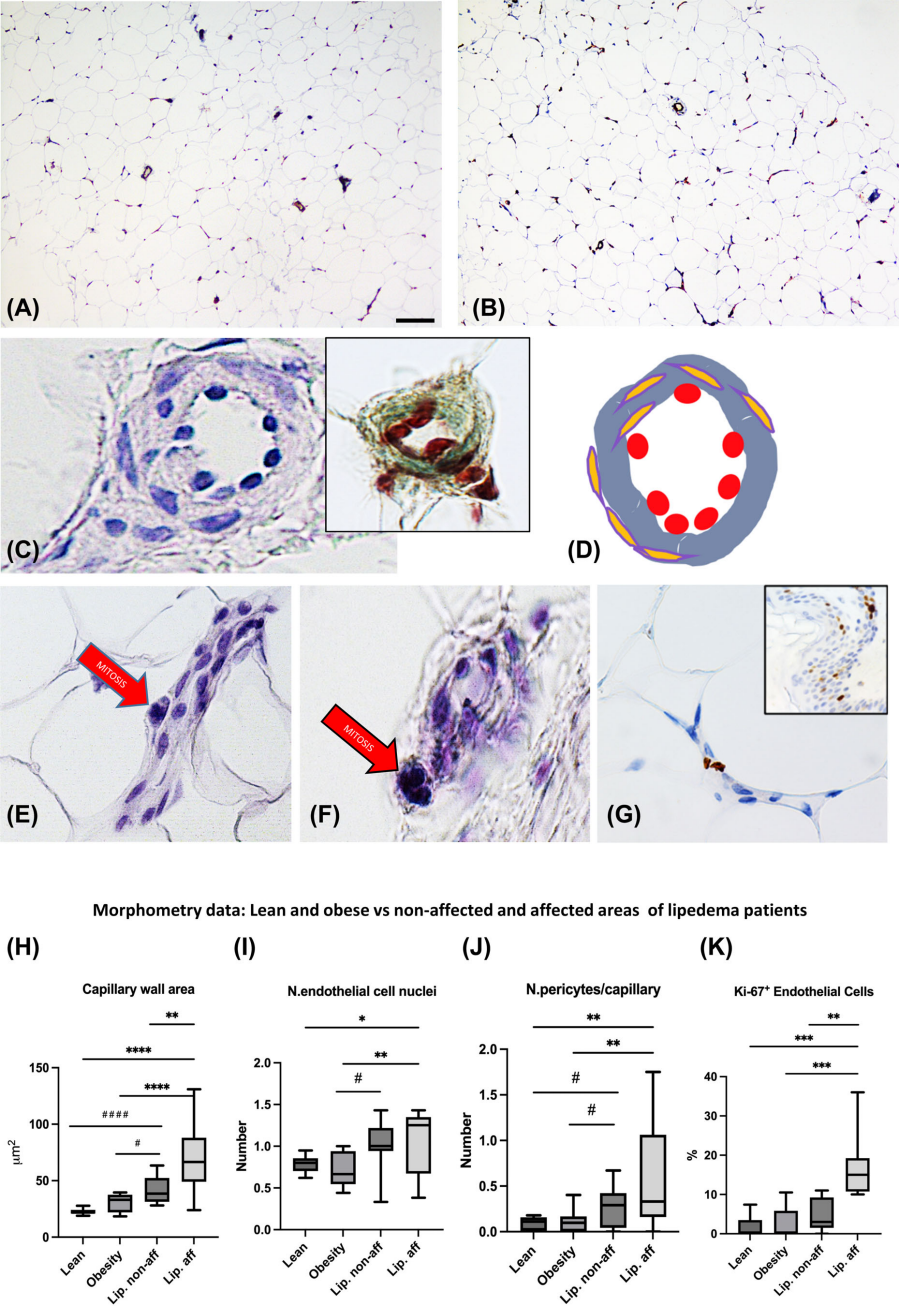
Representative light microscopy and morphometry data of subcutaneous adipose tissue of nonaffected and affected areas of patients with lipedema. (A,B) Small magnification (10×) of immunoreactivity of CD31 of nonaffected and affected areas, respectively. The hematoxylin & eosin staining shows high magnification (100×) of (C,F) affected and (E) nonaffected areas. Inset in panel C shows the Masson's trichrome stain confirming the thickened capillary wall. (D) Scheme of the capillary shown in panel C outlining the thickening of the basement membrane (blue), hyperplasia of endothelial cells (red), and pericytes (orange). (G) Representative immunostaining of Ki‐67 of affected area of a patient with lipedema. Inset in panel G shows immunostaining of Ki‐67 of a dermal area of the same patient bar: 20 μm in panels A and B; 7 μm in panel C; inset 10 μm and 15 μm in panel E; and 10 μm in panels F and G. Morphometry data: (H) capillary wall area: lipedema‐affected (vs. nonaffected *p* = 0.008, vs. obesity *p* < 0.0001, vs. lean *p* < 0.0001); lipedema nonaffected (vs. obesity *p* = 0.016, vs. lean *p* < 0.0001); obesity (vs. lean nonsignificant [n.s.] *p* = 0.56); (I) number of endothelial cell nuclei: lipedema‐affected (vs. nonaffected n.s. *p* = 0.78, vs. obesity *p* = 0.01, vs. lean *p* = 0.03); lipedema nonaffected (vs. obesity *p* = 0.03, vs. lean n.s. *p* = 0.1); and obesity (vs. lean n.s *p* = 0.81). (J) Number of pericytes per capillary: lipedema‐affected (vs. nonaffected n.s. *p* = 0.19, vs. obesity *p* = 0.008, vs. lean *p* = 0.004); lipedema nonaffected (vs. obesity *p* = 0.04, vs. lean *p* =0.02); obesity (vs. lean n.s. *p* = 0.9); (K) percentage of Ki‐67 positive endothelial cells: lipedema‐affected (vs. nonaffected *p* = 0.006, vs. obesity *p* = 0.0006, vs. lean *p* = 0.0001); and lipedema nonaffected (vs. obesity n.s. *p* = 0.3, vs. lean n.s. *p* = 0.2, obesity vs. lean n.s. *p* > 0.9). Statistical comparisons are represented by the pound symbol (#) for comparisons with lipedema nonaffected areas, whereas an asterisk (*) indicates comparisons with lipedema‐affected areas. [Color figure can be viewed at wileyonlinelibrary.com]

Our results showed, as a common characteristic feature of subcutaneous fat of areas affected by lipedema, a series of peculiar aspects of capillary vessels. The capillary vessels' walls were thickened and irregular. They showed hypercellularity due to increased endothelial as well as perivascular cells, and mitoses were often observed in the capillary wall (Figure 1A–F). Many endothelial cells were positive for Ki‐67 (Figure 1G).

**FIGURE 2:**
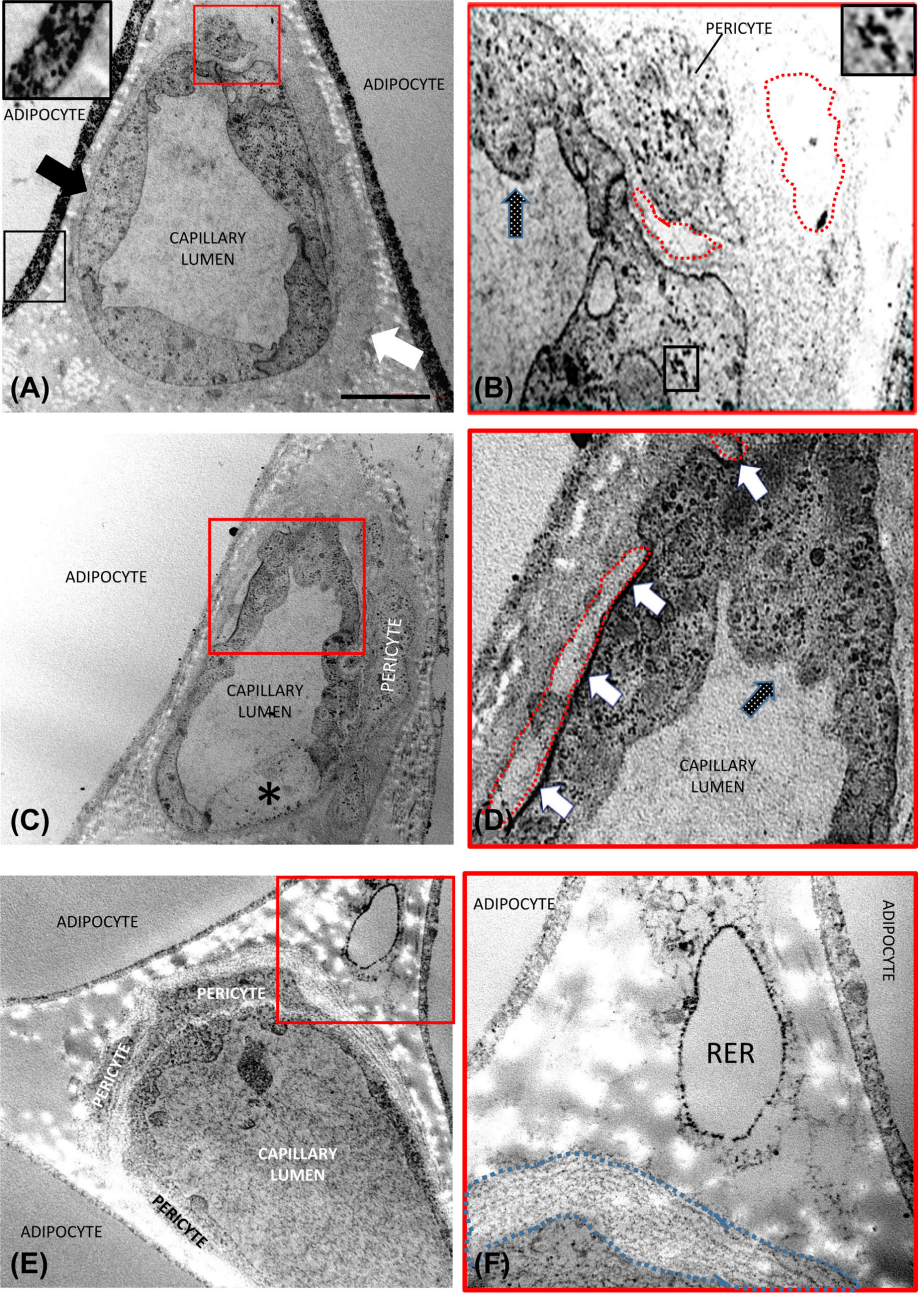
Representative electron microscopy of fat capillaries from affected areas of patients with lipedema. (A) Electron microscopy showing irregular thickening of the basement membrane (normal thickness is indicated by the black arrow; the thickened part is indicated by the white arrow). Framed area shows enlargement of calcium crystals in the adipocyte cytoplasm (less‐dense calcium deposits were seen also in endothelial cells, panels B and D). (B) Enlargement of the red framed area in panel A showing hypodense areas (dotted red line) of unknown nature. Note the dense particles due to the small amount of calcium crystals inside the cytoplasm of endothelial cells (inset: enlargement of framed area) and pericytes. (C) Capillary with focal increased density of the endothelial cell plasma membrane (framed area enlarged in panel D). Thickening of the basement membrane containing pericytes is visible. (D) In correspondence with plasma membrane hyperdensity, hypodense areas (white arrows) in the thickened basal membrane are visible (red dotted areas). (E) Capillary showing thickened and reduplicated basal membrane. Note the presence of three pericytes. (F) Enlargement of framed area in panel E. Reduplicated dense lines in the basal membrane are visible (blue dotted area). A poorly differentiated cell with dilated rough endoplasmic reticulum (RER), possibly representing a preadipocyte developmental stage is also visible. Many irregular cytoplasmic projections into the lumen were also visible in most of these endothelial cells (dotted arrows in panels B and D). Scale bar: 1 μm in panel A; 2 μm in panels C and E; 300 nm in panel B; and 650 nm in panels D and F. [Color figure can be viewed at wileyonlinelibrary.com]

**FIGURE 3:**
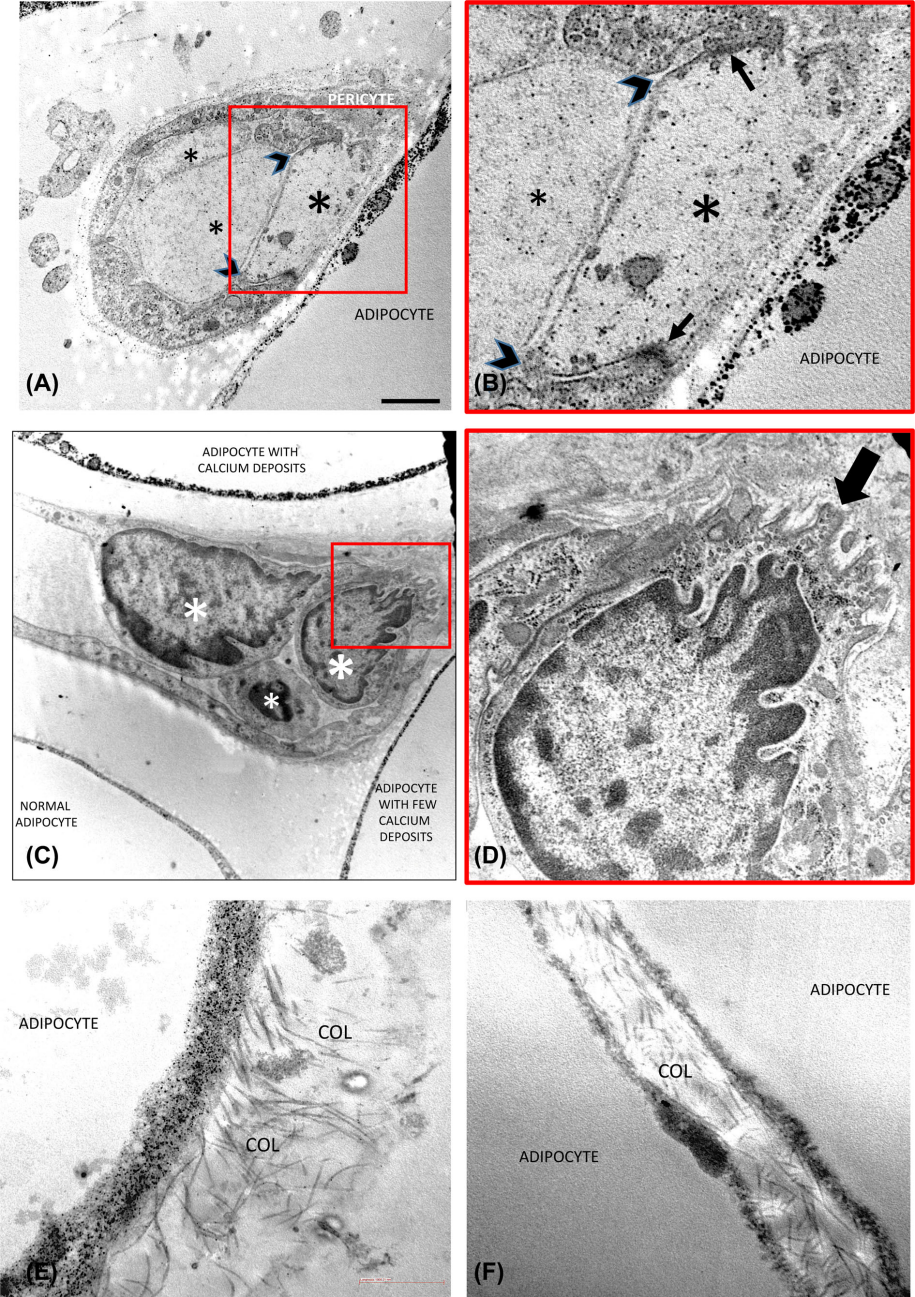
Representative electron microscopy of affected adipose tissue areas from patients with lipedema. (A,B) Capillary showing that one endothelial cell (large asterisk) is different from other endothelial cells because of its organelles’ rarefaction and hypodense cytoplasm; nevertheless, this cell is still joined by apparently normal junctions to other endothelial cells (thin black arrows), but the intercellular space was dilated (arrowheads). In the lumen two structures (small asterisks in panel A) with morphology similar to the degenerating endothelial cell (large asterisk in panel A) are visible. Note similar signs of endothelial cell degeneration marked by an asterisk in Figure 2C. (C) Electron microscopy showing a capillary among three adipocytes. Note the hypertrophic endothelial cells (large asterisks) and a cell in the lumen with ultrastructure similar to that of endothelial cells (small asterisk). Note the different quantity of calcium deposits in the cytoplasm of the adipocytes. (D) Enlargement of the square area in panel C showing an irregular connection with the basement membrane (arrow). (E,F) Visually evident increased collagen fibrils (COL) in close connection with the external adipocyte surface. Bar: 1 μm in panels A and C; 400 nm in panel B; 250 nm in panel D; 850 nm in panel E; and 500 nm in panel F. [Color figure can be viewed at wileyonlinelibrary.com]

**FIGURE 4:**
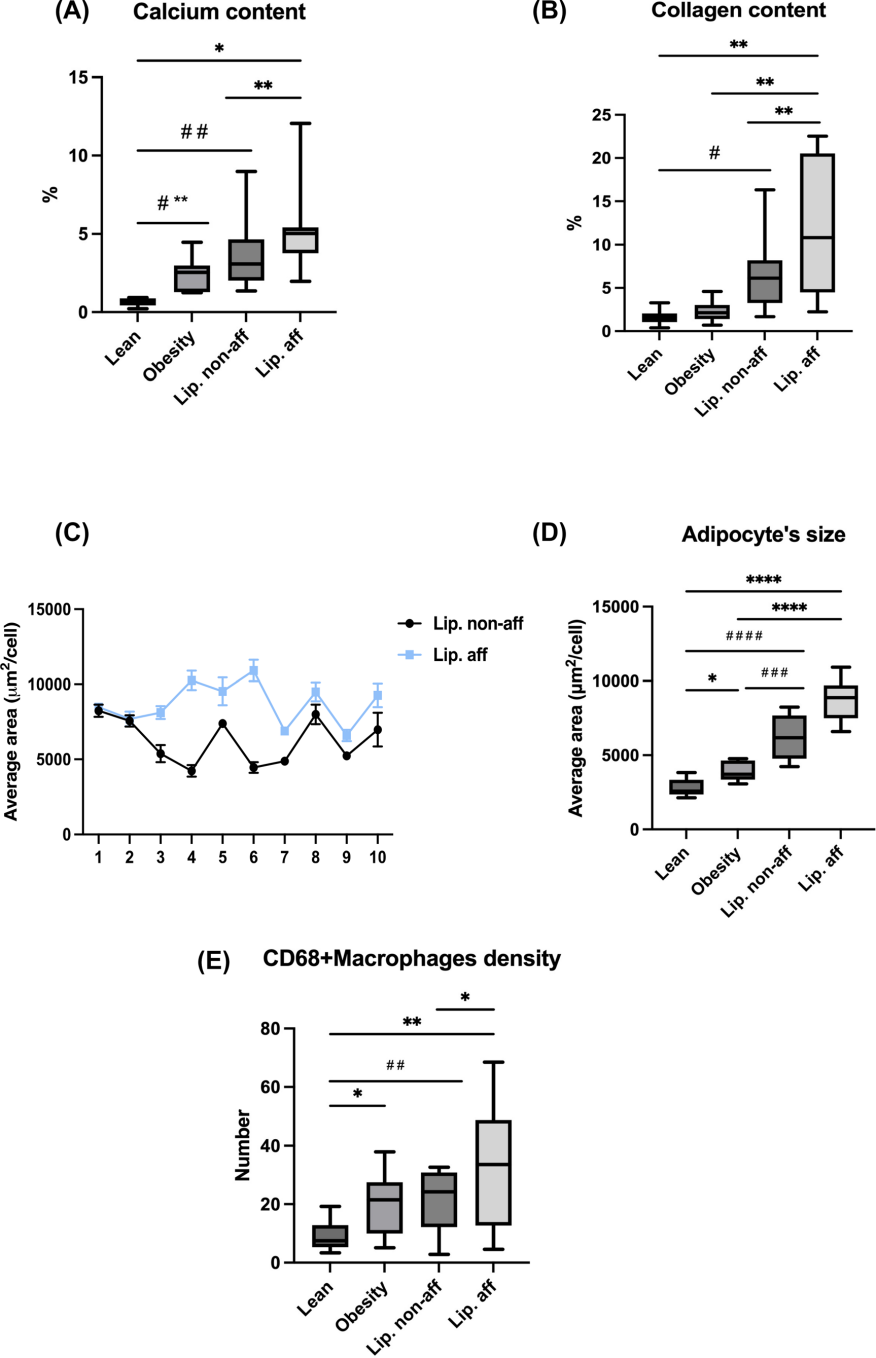
Morphometry data of adipose tissue. (A) Calcium content, as revealed by Von Kossa staining, showing higher prevalence in lipedema‐affected areas compared with both patients with obesity and lean patients, with the following significant *p* values: lipedema‐affected (vs. nonaffected *p* = 0.005, vs. obesity nonsignificant [n.s.] *p* = 0.05, vs. lean *p* = 0.02); lipedema nonaffected (vs. obesity n.s. *p* = 0.1, vs. lean *p* = 0.04); and obesity (vs. lean *p* = 0.007). (B) Collagen quantification showing higher prevalence in lipedema‐affected areas compared with both patients with obesity and lean patients, with the following significant *p* values: lipedema‐affected (vs. nonaffected *p* = 0.02, vs. obesity *p* = 0.009, vs. lean *p* = 0.002); lipedema nonaffected (vs. obesity *p* = 0.05, vs. lean *p* = 0.01); and obesity (vs. lean n.s. *p* = 0.3). Morphometric analysis of adipocyte size in (C) single patients with lipedema and (D) compared with patients with obesity and lean patients lipedema‐affected (vs. nonaffected *p* = 0.0052, vs. obesity *p* < 0.0001, vs. lean *p* < 0.0001); lipedema nonaffected (vs. obesity *p* = 0.0003, vs. lean *p* < 0.0001); and obesity (vs. lean *p* = 0.02). (E) Morphometric analysis of CD68 immunoreactive macrophages, showing higher density of CD68 immunoreactive macrophages in affected areas compared with nonaffected areas in patients with lipedema. Additionally, macrophages were more abundant in both affected and nonaffected fat compared with that in lean patients, although no significant difference was observed when comparing with patients with obesity. Statistical comparisons are represented by the pound symbol (#) for comparisons with lipedema nonaffected areas, whereas an asterisk (*) indicates comparisons with lipedema‐affected areas. [Color figure can be viewed at wileyonlinelibrary.com]

Comparing affected with nonaffected areas of patients with lipedema, the capillary wall area and the percentage of endothelial cells expressing Ki‐67 were greater in affected areas, whereas the number of endothelial and pericyte nuclei showed only a tendency to be increased (Figure 1H,I,J,K). When data were compared with control tissues from patients with obesity and/or lean patients, the capillary wall area, the number of endothelial cell nuclei, and the pericytes were significantly greater in patients with lipedema (Figure 1H,I,J). The affected areas from patients with lipedema had increased percentage of Ki‐67+ endothelial cells compared with both lean patients and patients with obesity (Figure 1K). When observed by TEM (Figures 2, 3, 4), these vessels showed the following: 1) irregular profile of endothelial cells with cytoplasmic protrusions in the capillary lumen (Figure 2B,D); 2) thickened (Figure 2A–D) and sometimes reduplicated (Figure 2E,F) basal membrane with hypodense irregular areas of unknown nature (Figure 2B,D); 3) hyperdensity of invaginated portions of endothelial plasma membrane often closely associated to hypodense areas of basal membrane (Figure 2C,D); and 4) evident signs of endothelial cell degeneration either continuing as part of the capillary wall or exfoliated in the capillary lumen (Figure 2C and Figure 3A,B). In agreement, immunohistochemistry with the endothelial‐specific antibody marker CD31 showed focal absence of staining in several capillaries (Figure S1), including visually evident dilatation of the intercellular space between the degenerating and normal endothelial cells (Figure 3A,B); in line with these alterations, we also found signs of endothelial cell detachment from the capillary wall (Figure 3C,D).

The aforementioned endothelial alterations were frequently found mainly in affected areas of patients with lipedema but never in subcutaneous fat of patients with obesity or lean patients (Figure S2). Several alterations were also found in parenchymal and interstitial components of the tissue. Adipocytes showed abundant cytoplasmic calcium crystals (Figure 2A,E and Figure 3B,C); Von Kossa staining confirmed the nature of calcium crystals (Figure S3). Morphometric analysis revealed a significant increase in the percentage of calcium deposition in affected areas compared with nonaffected areas of patients with lipedema (*p* = 0.005). Both areas of patients with lipedema showed higher amounts of calcium compared with lean controls, i.e., *p* = 0.04 and *p* = 0.02, respectively (Figure 4A).

Less‐abundant calcium crystals were present also in endothelial cells (Figure 2B,D).

Visually evident increased deposition of collagen fibers (thickness of 25–40 nm) among adipose cells was a constant feature in affected areas of patients with lipedema, as well as in patients with obesity, but not in lean patients (Figure 3E,F). Morphometric analysis of Masson's trichrome staining demonstrated a significant increase in collagen deposition (fibrosis) in affected areas compared with nonaffected areas of patients with lipedema (*p* = 0.02). Patients with lipedema revealed more collagen content compared with lean controls in affected (*p* = 0.002), as well as in nonaffected, areas (*p* = 0.001). The affected areas of patients with lipedema were also more fibrotic than that of patients with obesity (*p* = 0.009; Figure 4B).

Rarely, elastic fibers were also present among adipocytes only in affected areas of patients with lipedema (not shown). D2‐40 immunoreactive vascular structures, markers of lymphatic vessels [14, 15, 16], were not identified in any of the studied specimens.

Adipocytes in affected areas were more hypertrophic than adipocytes of nonaffected areas in eight out of ten patients with lipedema. Adipocytes of both affected and nonaffected areas from patients with lipedema were significantly larger compared with adipocytes from lean patients and patients with obesity (Figure 4C,D). Given the well‐established links among adipocyte size, cell death, and inflammation [17, 18], we quantified CD68‐positive macrophages, the predominant inflammatory cells in adipose tissue of individuals with obesity. CD68‐positive macrophages were more abundant in affected areas compared with nonaffected areas in patients with lipedema, consistent with the increased adipocyte size in affected tissue [19]. Macrophage presence was significantly higher in patients with lipedema than in tissue from lean patients but was similar to that in tissue from patients with obesity (Figure 4E), indicating that inflammation in lipedema‐affected fat resembles that found in the presence of obesity.

Perilipin is a protein crucial for the normal activity of adipocytes. Our previous work suggested that its absence is a sign for unhealthy tissue conditions, and its absence in hypertrophic adipocytes from patients with obesity is considered as a marker of death [18, 20].

Surprisingly, perilipin was rarely absent in both affected and nonaffected areas in tissue from patients with lipedema, suggesting that, despite their hypertrophic size, adipocytes from lipedema patients are less prone to the unhealthy state of hypertrophic adipocytes from patients with obesity. Similarly, CLS, which are histopathological indicators of dead adipocytes [18], were infrequently observed in tissue from patients with lipedema. In fact, CLS were found in the subcutaneous fat of two out of twelve lean patients, five out of ten patients with obesity, and only one out of ten in the adipose tissue of patients with lipedema. Remarkably, none of the fat biopsies from the nonaffected areas of patients with lipedema showed any evidence of CLS.

## DISCUSSION

Although lipedema is a quite common disease affecting mainly adult female individuals, its pathogenesis remains obscure [1]. Previous and recent work has pointed to alterations of vasculature [21, 22, 23, 24]. Our data, although obtained only via an observational study, provide some support for the involvement of vascular changes in this pathology.

About 10 years ago, we published data supporting the idea that the stem cell of origin of adipocytes in mice is the endothelial cell of adipose tissue [25, 26]. We are aware that not all the scientific community agrees with our interpretation; however, there are numerous published data to support our opinion, including the following: 1) isolated mature human adipocytes dedifferentiate into endothelial‐like cells [27, 28], and endothelial cells can be converted into mesenchymal stem cells that can differentiate into adipocytes [29]; 2) capillary networks and single‐cell suspensions from microvessels of human fat explants give rise to well‐characterized adipocyte progenitors able to develop into mature adipocytes in a cell‐autonomous manner [30]; 3) perilipin, adiponectin, and preadipocytes were found to emerge at embryonic day 16.5 in inguinal white adipose tissue and proliferated to form clusters strongly interacting with growing adipose vasculature and endothelial‐specific Vegfr2 depletion‐induced vascular disruption with interruption of in vivo adipogenesis [31]; 4) the endothelial origin would imply endothelial‐mesenchymal transition that is linked to bone morphogenetic proteins/transforming growth factor β signaling and absence of the downstream effector myocardin‐related transcription factor A seems to induce commitment of progenitors to adipogenesis [32]; and 5) capillary endothelial cells of adipose tissue express the adipogenic commitment factor ZFP423 [26].

Therefore, the endothelial and pericyte hyperplasia found here, mainly in adipose tissue of affected areas of patients with lipedema, is in line with the idea that these cells, developing into adipocyte precursors, give rise to hyperplastic abnormal adipose tissue development that is an evident pathogenetic event and clinical sign of this disease. Furthermore, the altered morphology of capillaries fits with the possibility that the development of adipocytes from altered precursors could give rise to altered mature adipocytes. As a matter of fact, adult adipocytes were often abnormal in that they were hypertrophic with intact perilipin and often rich in calcium crystals. Of note, calcium crystals have been previously identified in adipocytes from patients with obesity [30], which was confirmed in the present work in which quantitative data demonstrated a significant increase of calcium both in affected and nonaffected fat from patients with lipedema when compared with lean control patients (*p* = 0.04; *p* = 0.02). Of note, hypertrophy of lipedema adipocytes was higher than that found in patients with obesity despite the normal body mass index (BMI) of the patients with lipedema studied herein, further suggesting a cell‐autonomous alteration in adipocyte precursors and derived mature cells. In the present study, we found that hypertrophic adipocytes from patients with lipedema, mainly in affected areas, were infiltrated by CD68 macrophages, but CLS, quite frequently observed in patients with obesity, were rare. Consistent with our findings, lipedema has been characterized by an increased population of M2‐polarized macrophages (referred to as “alternatively activated”) and a state of low‐grade inflammation [33], along with elevated levels of macrophage migration inhibitory factor [34]. In contrast, the adipose tissue of patients with obesity typically exhibits a classical inflammatory state mainly composed of M1 polarized macrophages (defined as “classically activated”) [35].

This aspect of lipedema tissue can be explained by the altered nature and development of these adipocytes, which allowed for a much larger size in the adipocytes from lipedema patients before a critical death size was reached [36, 37]. Supporting this hypothesis, perilipin was present in the vast majority of adipocytes, including the largest ones, suggesting that these much larger lipedema adipocytes were still in good health. Most of the aforementioned alterations were also observed in nonaffected areas even if quantitative data demonstrate a significant difference. Collectively, these findings suggest that lipedema induces a global involvement of the entire adipose organ, with specific localized areas exhibiting more pronounced effects. The gender‐specific topographical expression of female sex hormone receptors further supports the role for these hormones in the pathogenesis of lipedema, wherein endothelial cells express receptors for steroid hormones, including progesterone [38, 39]. Notably, a mutation in the gene responsible for the synthesis of aldo‐keto‐reductase in a family with nonsyndromic primary lipedema has been described, suggesting that the inability to degrade progesterone may contribute to lipedema pathology [40]. Interestingly, progesterone influences vascular structures and other cell types via calcium channels, potentially linking it to the accumulation of calcium crystals that we observed in adipocytes and vascular cells [41]. Some fine alterations identified here, including the endothelial cell membrane hyperdensity concomitant with circumscribed basal membrane alterations, were unique to the affected areas of patients with lipedema and were never found in patients with obesity or lean patients. These findings warrant further investigations to reveal the molecular consequences of such changes.

The observed endothelial hyperplasia, along with signs of endothelial degeneration and vascular invasion, could provide a new, noninvasive diagnostic tool for lipedema. Intriguingly, the endothelial cell marker Ve‐cadherin has already been detected in the blood of cardiac patients, where it was found at higher levels than in controls [42]. The alterations described in the present work predict that it could be significantly elevated in patients with lipedema, potentially serving as a novel and early blood marker of lipedema.

The absence of any sign of lymphatic alteration in the tissue studies here may be attributed to the early stage of lipedema of the patients examined, aligning with findings from other studies [43].

Our observations of Ki‐67^+^ endothelial cells and frequent mitotic activity within the capillary walls, supported by quantitative data indicating the proliferation of endothelial cells and pericytes, are suggestive that these locations are the source for new adipocytes. This aligns with previous studies [25, 26] and recent findings linking Bub1 gene dysregulation to increased proliferation [44] and angiogenesis [21] in the adipose tissue of patients with lipedema.

Regarding limitations of the present study, we must take into account two different aspects. First, it is necessary to consider the small number of patients that we analyzed. This was mainly due to a strict selection to narrow the study to patients in the early stages of lipedema in order to avoid confounding histopathological aspects. Additionally, this investigation is not exhaustive in terms of examined aspects and methodologies employed. The data presented here are primarily derived from histology, ultrastructural morphology, immunohistochemistry, and morphometry, as we focused on understanding the cellular physiopathology of this disease.

The biopsies analyzed in this study were all from subcutaneous fat but were taken at different anatomical localizations. Specifically, biopsies from affected areas of lipedema patients were collected from the limbs, compared with an expected unaffected area in the interscapular region [1, 8]. For ethical reasons, it was not feasible to perform biopsies at the same site in control patients with obesity. The lean control group consisted of patients undergoing cholecystectomy who had been confirmed to have a healthy metabolic profile [45]. Overall, our findings deserve more extensive investigations involving a larger cohort of patients and controls, incorporating molecular and functional assessments.

## CONCLUSION

Our data suggest a potential role for endothelial cell alterations in the pathogenesis of lipedema. The evident proliferation of these cells, along with increased pericyte density, may contribute to the development and the proliferation of lipedema adipose tissue, considering the developmental data from our and other's studies [17, 18]. The alterations of endothelial cells revealed in this study by electron microscopy provide an explanation for pathological hypertrophy of adipocytes with calcium metabolism alterations, triggering macrophage infiltration. Because calcium channels are hormone‐ and cytokine‐dependent, a redundant abnormal pathogenetic circuit cannot be excluded. Interestingly, the stromal‐vascular fraction of lipedema tissue is characterized by a distinct cytokine profile and metabolic activity [46]. Therefore, a role for cytokines additionally in the induction of the characteristic pain associated with lipedema tissue can be hypothesized.

## AUTHOR CONTRIBUTIONS

Sandro Michelini, Stefania Greco, Karen L. Herbst, Antonio Giordano, Pasquapina Ciarmela, and Saverio Cinti: study conceptualization; Sandro Michelini, Antonio Giordano, Pasquapina Ciarmela, and Saverio Cinti: study coordination; Sandro Michelini, Nicola Vaia, Valeria Puleo, and Serena Michelini: collection of bioptic samples and clinical data; Stefania Greco, Pamela Pellegrino, Angelica Di Vincenzo, Gaia Goteri, Tonia Luca, Sergio Castorina, Antonio Giordano, Pasquapina Ciarmela, and Saverio Cinti: histological studies; Stefania Greco, Pamela Pellegrino, Pasquapina Ciarmela, and Saverio Cinti: electron microscopy studies; Sandro Michelini, Stefania Greco, Pamela Pellegrino, Angelica Di Vincenzo, Karen L. Herbst, Gaia Goteri, Tonia Luca, Sergio Castorina, Antonio Giordano, Pasquapina Ciarmela, Karen L. Herbst, and Saverio Cinti: data analyses and interpretation. All authors approved the final version of the manuscript and take responsibility for its content.

## CONFLICT OF INTEREST STATEMENT

The authors declared no conflicts of interest.

## Supporting information


**Figure S1.** Representative immunoreaction of CD31 in affected areas of patients with lipedema. The arrow heads indicate the focal absence of staining in endothelial cells of capillaries. Bar: 5 μm in all.
**Figure S2.** Representative electron microscopy of adipose tissue capillaries from control patients with leanness, in nonaffected areas of patients with lipedema and in patients with obesity. All capillaries show no sign of alterations and regular and thin basement membranes.
**Figure S3.** Representative Von kossa stain of subcutaneous fat from affected areas of lipedema patients. The arrows indicate the black staining due to the presence of calcium crystals. Bar:7 μm in all.

## Data Availability

The datasets used and analyzed during the current study are available from the corresponding author on reasonable request.
