# Supplementary material for: Endothelial cell alterations in capillaries of adipose tissue from patients affected by lipedema
Source: Obesity (Silver Spring). 2025 Mar 12;33(4):695–708. doi: 10.1002/oby.24244 (PMC11937865; doi:10.1002/oby.24244)
Supplement: Supplementary file 1 — Figure S1. Representative immunoreaction of CD31 in affected areas of patients with lipedema. The arrow heads indicate the focal absence of staining in endothelial cells of capillaries. Bar: 5 μm in all. Figure S2. Representative electron microscopy of adipose tissue capillaries from control patients with leanness, in nonaffected areas of patients with lipedema and in patients with obesity. All capillaries show no sign of alterations and regular and thin basement membranes. Figure S3. Representative Von kossa stain of subcutaneous fat from affected areas of lipedema patients. The arrows indicate the black staining due to the presence of calcium crystals. Bar:7 μm in all. [file OBY-33-695-s001.pptx]

## Slide 1
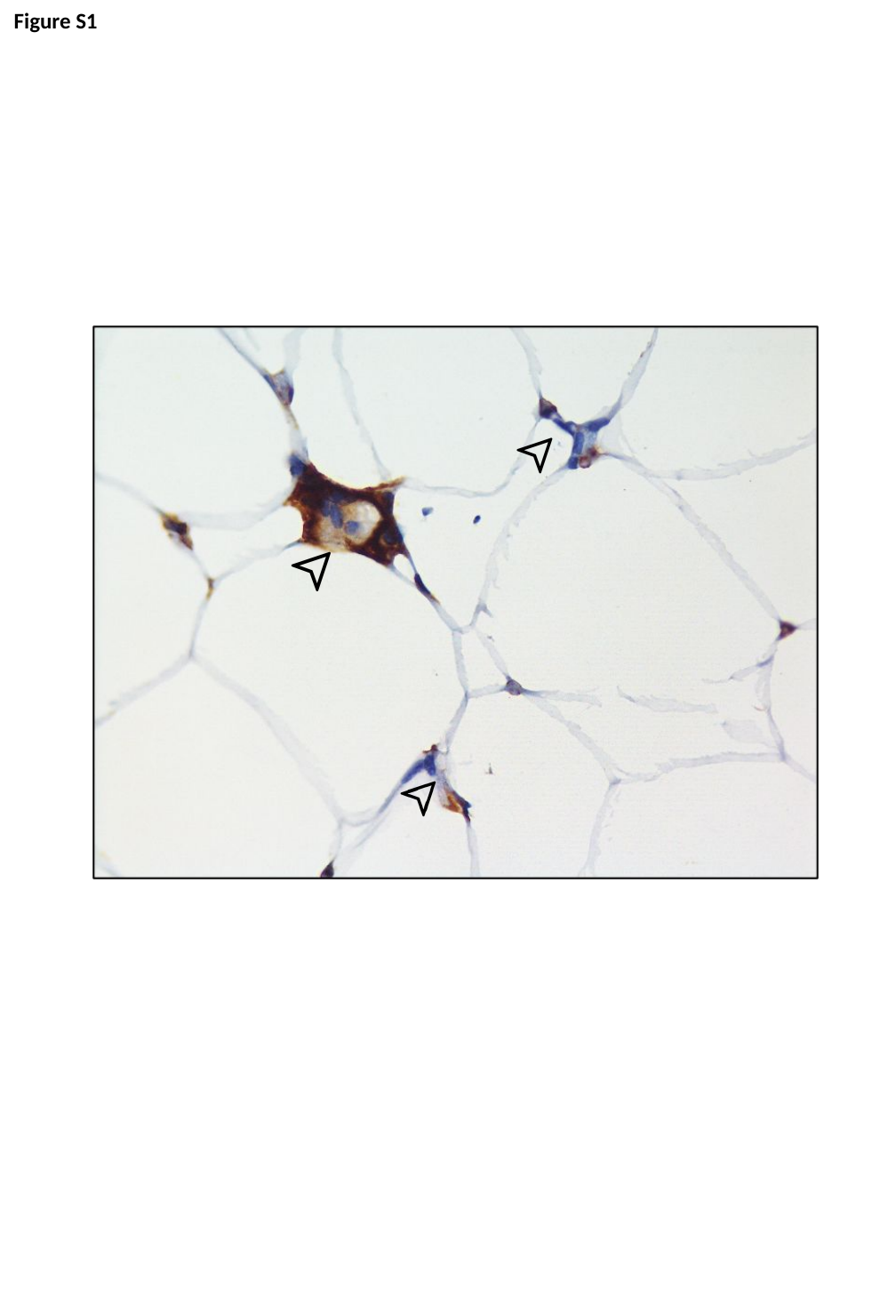

Figure S1

## Slide 2
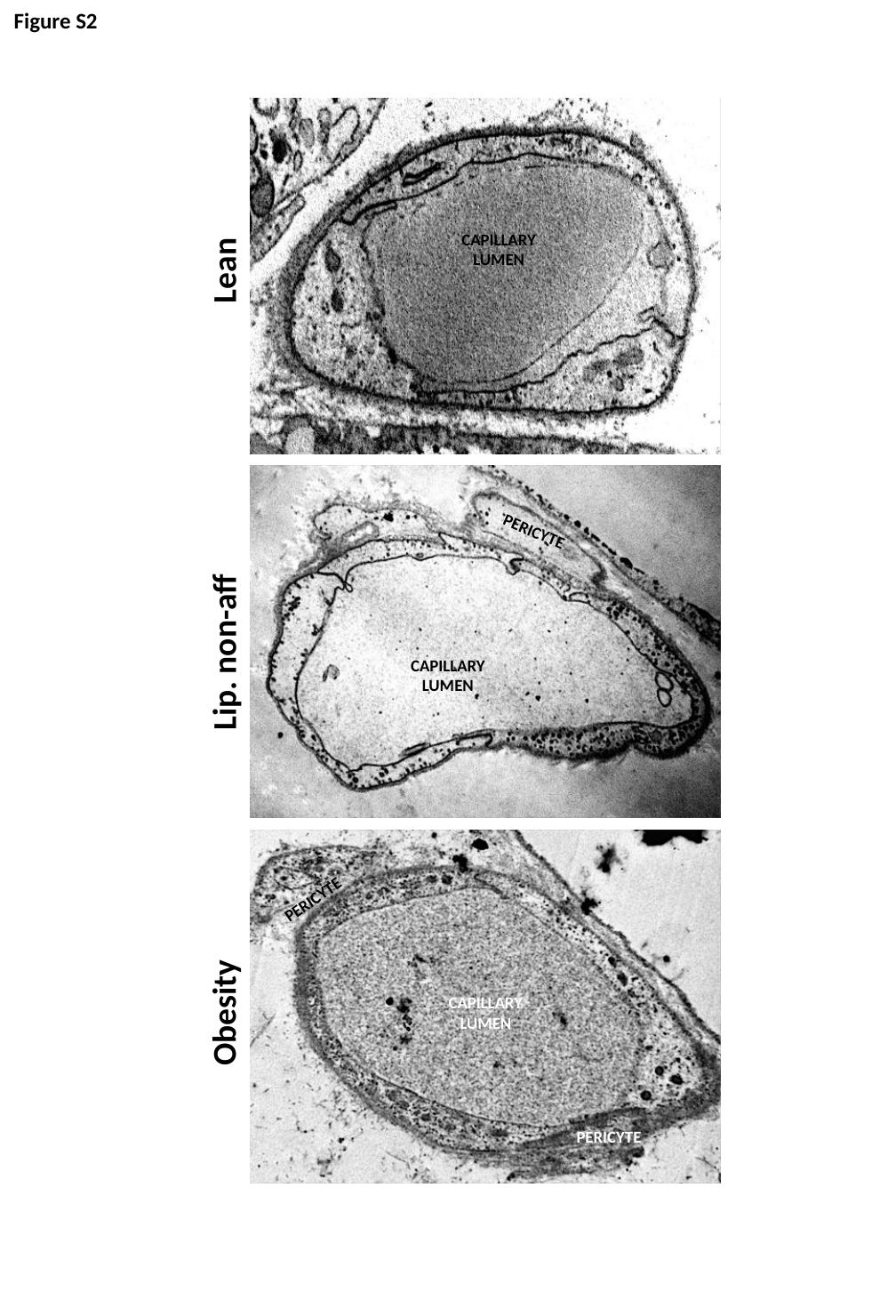

Figure S2
CAPILLARY
LUMEN
Lean
PERICYTE
Lip. non-aff
CAPILLARY
LUMEN
PERICYTE
Obesity
CAPILLARY
LUMEN
PERICYTE

## Slide 3
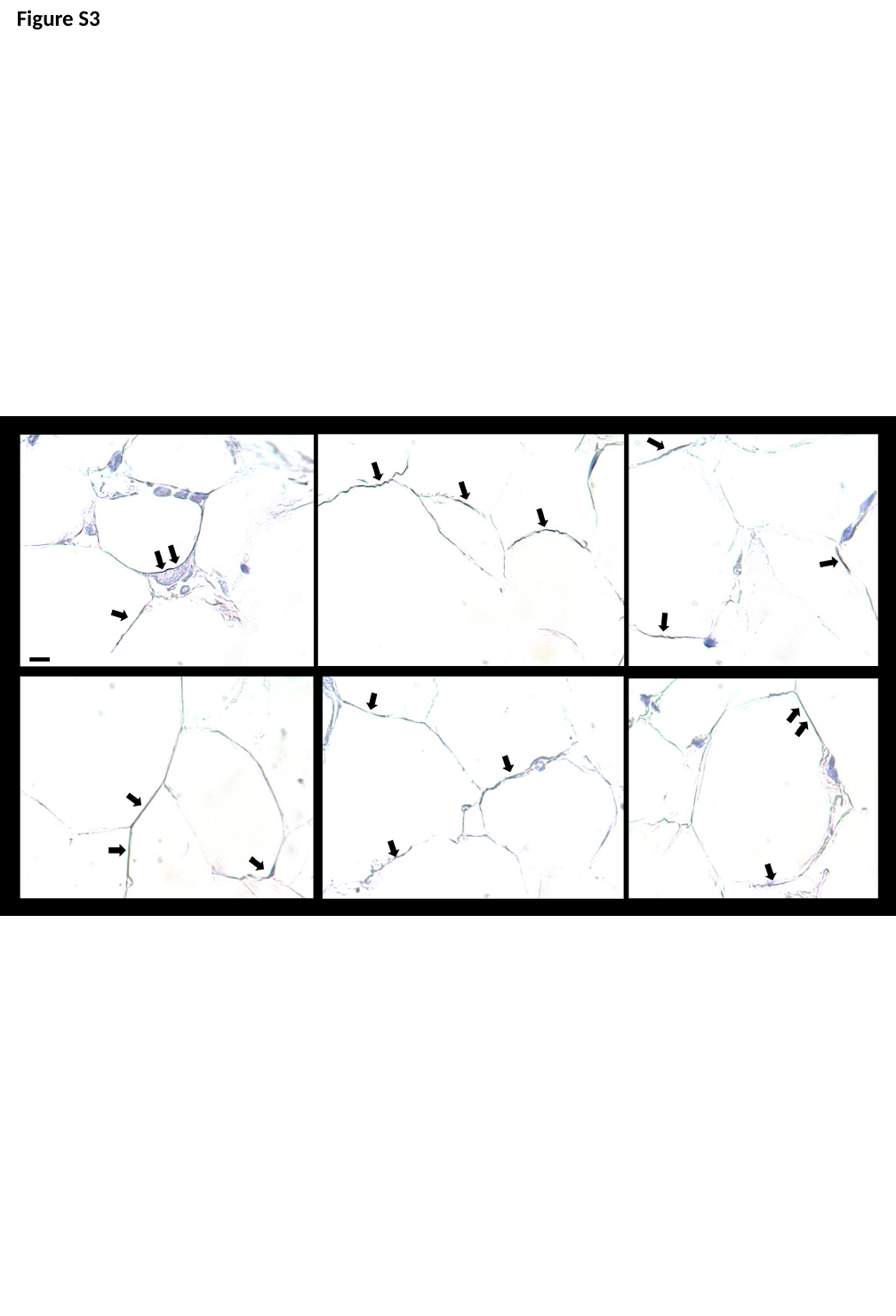

Figure S3
